# Child Protection, Disability and Obstetric Violence: Three Case Studies from Iceland

**DOI:** 10.3390/ijerph18010158

**Published:** 2020-12-28

**Authors:** James Gordon Rice, Helga Baldvins Bjargardóttir, Hanna Björg Sigurjónsdóttir

**Affiliations:** 1 School of Social Sciences, University of Iceland, 105 Reykjavík, Iceland; hbs@hi.is; 2 HelgaBaldvins slf, 103 Reykjavík, Iceland; helga@helgabaldvins.is

**Keywords:** disability, Iceland, child protection, obstetric violence, custody deprivation, intellectual disability, disability studies

## Abstract

This contribution is a collective re-analysis of three research projects in Iceland focused on parenting with a disability which draws upon data spanning a twenty-year period. The core purpose of these projects is to understand why parents with primarily intellectual disabilities encounter such difficulties with the child protection system. Our aim with this contribution is to identify, through a longitudinal and comparative framework, why these difficulties persist despite a changing disability rights environment. A case study methodology has been employed highlighting three cases, one from each research project, which focus narrowly on disabled parents’ struggles with the child protection system in the context of the maternity ward. The findings, framed in the concept of structural violence, indicate poor working practices on the part of healthcare and child protection, a lack of trust, and that context is still ignored in favour of disability as the explanatory framework for the perceived inadequacies of the parents. We contend that child protection authorities continue to remain out of step with developments in disability and human rights. The contribution concludes to make a case as to why the concept of obstetric violence is a useful framework for criticism and advocacy work in this area.

## 1. Introduction

This contribution is a collective re-analysis of three research projects in Iceland focused on parenting with a disability which draws upon data spanning a twenty-year period. The cases selected from this body of work share commonalities in that they concern parents, or a parent, with an intellectual disability. They share the status of being the subject of child protection interference. Further, the child protection involvement began in the context of, or shortly before, the maternity ward setting. The first research project (Family support services and parents with learning difficulties 2001–2005) is the doctoral project of the third author [1] which was an ethnographic study of parents with intellectual disabilities focusing on the stories of eight families. The second research project (Family life and disability 2014–2016) analysed a national sample of custody deprivation cases in Iceland from 2002 to 2014, with a smaller select sample pursued up to 2016 with a particular focus on intellectual disability. The third research project (Disability, immigration and multigeneration: intersecting factors in child protection cases 2020–2022) is ongoing, with preliminary findings from one case presented here. The legal and human rights environment has changed significantly in Iceland since the beginning of the 21st century, as seen in part with Iceland’s signing the United Nations Convention on the Rights of Persons with Disabilities (CPRD) in 2007. However, it took nearly a decade for Iceland to ratify the Convention. The questions that motivated this contribution sought to understand if there were commonalities across the temporal span of these research projects. A second goal was to consider what, if any, differences in the working practices of child protection were evident, and if so if they could be linked to the changing legal and human rights environment in Iceland over the last two decades.

### 1.1. Background and Theoretical Framework—Disability and Child Protection

Previous evidence indicates that the children of parents with intellectual disabilities have been removed from their parents’ care at disproportionate rates compared with other parents [2,3,4]. Evidence is lacking which demonstrates a clear correlation between IQ (intelligence quotient) level and parenting ability [5], and that with proper and tailored support parents with intellectual disabilities (ID) can provide adequate care [6,7]. When parents with ID come into contact with child protection, custody termination is not an unusual outcome. In such cases evidence of abuse or neglect is either lacking or the reference to disability or low IQ acts as a form of proxy evidence of the risk of abuse or neglect. Once a child protection (CP) case has been opened, it is exceedingly difficult to prevent an outcome of custody deprivation, even if the accusations of neglect have been refuted or the parents have demonstrated progress in support programmes and have co-operated with the child protection authorities. Common interpretations for these outcomes point to discriminatory attitudes and stigmas about disability, and intellectual disability in specific, which hold the disabled parents across the board are incapable or incompetent in the parenting role [8,9]. Research points to the factors of improper or insufficient support and lack of training on the part of CP and support staff [10,11,12,13,14] and flawed or lacking disability assessments during CP investigations [15]. Deficiencies in regard to CP investigation techniques, parenting capacity and risk assessment procedures have been noted in general [16] and which are intensified in the context of parents with ID [9,17,18,19,20].

### 1.2. Structural and Obstetric Violence

We have also drawn inspiration from the sociologist Johan Galtung’s work [21] on structural violence. While Galtung does not specifically consider disability, his triangle of violence is a helpful framework in which he posits that direct violence is equally dependent upon structural violence as well as cultural violence. Direct violence, such as an act of forcefully removing a child from its birth parents, is only comprehensible if one considers the context of indirect structural violence that allows these acts to occur (e.g., institutionalised forms of disablism, sexism, racism, labour exploitation, etc.) and the ideologies and discourses of cultural violence which informs, legitimates and justifies these violent outcomes. Galtung’s observation that structural violence “*is built into the structure and shows up as unequal power and consequently as unequal life chances*” ([21], p. 171) for us aptly articulates the processes and the effects upon outcomes that we have observed in cases where parents have unfairly lost the custody of their children and have been denied the possibility of being parents on the basis of disability.

Our focus on interpreting the removal of children from parents on the basis of disability as structural violence also led us to consider the body of work that advances the concept of obstetric violence. The concept of obstetric violence has increasingly entered into the vocabulary of legal systems in the early 21st century since its first introduction in Venezuela in 2007 [22]. The small but growing literature on obstetric violence shares a focus on some common issues, such as the over-medicalisation of the birth process (a pathological rather than natural view of the birth process; excessive or unnecessary use of drugs or other medical procedures), harsh treatment (forceful or aggressive handling of the body by staff; negative comments made to mothers; value judgements) and issues of consent (the use of medical procedures during the birthing process without full and informed consent), which are concerns that have been raised by international organizations such as the World Health Organization since the 1980s [23]. A common theme within this literature is the desire to articulate the negative experiences of women concerning the birth processes as violence, rather than mistreatment or malpractice, and to include within the analysis issues often dismissed as trivial or non-violent, such as dehumanization, loss of autonomy and discursive forms of violence discussed by some scholars as microaggressions [24]. Chadwick [25] argues that the “*use of the controversial term ‘obstetric violence’ over more neutral labels such as ‘mistreatment’ is part of a deliberate move to confront problematic practices, which have often been hidden, invisible and unacknowledged, as forms of violence*” ([25], p. 423). Galtung concedes that social injustice could be used synonymously with structural violence [21] (p. 171), but as Chadwick suggests the use of the term violence is intentional in order to articulate a level of gravitas that may be less apparent with a term like injustice or discrimination. Obstetric violence appears to be directed to a higher and more intense degree toward marginalized women in regard to class, rural status, and race or ethnic minority/indigenous status [24,25,26,27]. The factor of physical disability does appear in the small literature on obstetric violence and disability [28,29], but much less so intellectual disability.

### 1.3. Disability and Human Rights

An important background to our research projects, and this contribution in particular, is the changing social and legal environment. Icelandic disability legislation in the latter part of the 20th century generally reflected Nordic, European and international developments in the area of services as well as human rights [1,30]. This can be seen with important milestone legislation in Iceland that reflected the rising influence of the ideology of normalization, deinstitutionalization and community living in the 1960s and 70s [30]. However, this growing awareness of the equality issues faced by disabled people did not extend toward the area of parenting. No legislation in Iceland during this time referred to the right of disabled people to be parents ([1], p. 14). Arguably, the ideological legacy in Iceland that governed the approach to parenting with a disability, particularly intellectual disabilities, is the eugenics movement that was dominant in the Nordic countries in the early to mid-part of the 20th century [1]. Preventative measures such as sterilization and sex-segregated institutions were utilized as a way in which to prevent people with intellectual disabilities from becoming parents. This eugenic inspired approach remained dominant in Icelandic legislation and practices until the 1970s and 80s ([9], p. 79), yet traces of this thinking can still be found in the present. For example, the current working definition of neglect by the child protection authorities in Iceland includes leaving a child in the care of someone with ID [31].

Iceland signed the United Nations Convention on the Rights of Persons with Disabilities in 2007 and ratified the Convention in 2016. The CRPD is rather broad in scope and Theresia Degener contends: “*It is the first human rights instrument which acknowledges that all disabled persons are right holders and that impairment may not be used as a justification for denial or restrictions of human rights*” ([32], p. 1). Leslie Francis [33] argued that Article 23 of the CRPD (Respect for home and the family) and Article 12 (Equal recognition before the law) are of particular importance for parents with ID. Article 23 holds that State Parties are required to eliminate discrimination in the area of the family and parenthood, ensuring that disabled people are able to maintain guardianship of children. Francis continues that it is Article 12 that provides a legal model as to how this can be achieved, as Article 12 touches directly on the key challenges faced by parents with ID in the context of child protection cases: the right to equal recognition before the law, their right to enjoy legal capacity on the basis of others, and the provision of access to support to ensure that they can exercise their legal capacity [33] (p. 22). Article 5 specifies reasonable accommodation as an obligation necessary to achieve equality and that the lack thereof constitutes discrimination: “*This clearly demonstrates that reasonable accommodation is not conceived as an exception to the prohibition of discrimination, but as an intrinsic element of the duty of equal treatment*” ([34], p. 7).

## 2. Methods

The cases presented here for re-analysis have been selected and drawn from three research projects in Iceland. The methodology of each project is somewhat different, as is the time period in which the research took place. However, we are pursuing a re-analysis of these cases as a collective in order to examine why parents with intellectual disabilities continue to struggle with the child protection system despite a changing human rights environment. The specific methodology and a brief description of the context of each case follows, after which our collective case study methodology for this contribution is detailed.

### 2.1. Case Study One: Stuck in the Hospital

The first case is drawn from the project Family support services and parents with learning difficulties. The data were collected over the period of 2001 to 2003. The first stage of the project involved interviews with a sample of eight families that included parents with intellectual disabilities to collect and analyse their views on the support and services they received. The parents were recruited as random participants through five social services agencies, satisfying the criteria that they had children under the age of 16 and were receiving support from their municipality. The second stage involved focus group interviews with those who provided support to the families, followed by participant observation in the home where services were provided, and concluding with an analysis of relevant documents written about the families during the time of the study.

The case presented here concerned a couple with intellectual disabilities in their mid to late 20s at the time. When the woman became pregnant (first child), social services were contacted by the expectant mother’s mother as the larger family on both sides knew the couple would need support. None of the family members anticipated that their request for support would ultimately lead to permanent custody deprivation of the couple’s child. The third author conducted interviews with all relevant parties (parents, extended family, maternity ward staff, social services, child protection, legal professionals, among others) after the couple lost custody of their new-born child.

### 2.2. Case Study Two: The Multigeneration Effect

The events of the second case presented here occurred roughly a decade later and was analysed within the project Family life and disability (2014–2016). The core dataset was a national sample of custody deprivation cases in Iceland from 2002 to 2014 (n. 57), with a smaller select sample pursued up to 2016 with a particular focus on ID. The project was an exercise in critical discourse analysis [35], but employed at the start a grounded theory approach inspired by Glaser and Strauss [36] with later insights provided by Charmaz [37]. The original intent was to compare and contrast what, if any, differences were evident in how custody deprivation cases were worked through comparing cases that concerned disabled parents with other cases. The cases were read line-by-line in sessions by the first and third authors, taking notes, constructing timelines of events, and comparing and contrasting cases. Through this process we constructed codes out of which we built thematic analyses which were in turn applied to selected cases. This resulted in a number of publications, each of which reflected one of our major thematic analyses such as stigma [9], case evidence construction [38], and the use of notifications [17].

The case presented here concerned two parents with ID. They had been together over a decade and, at the time, had a stillborn child the year earlier before the pregnancy that is the focus here. As with the prior case, a notification to CP was made concerning fears by healthcare staff about the couple’s child rearing abilities as the result of their impairments. In this case both parents had a history of interference in their lives from child protection since they were themselves children.

### 2.3. Case Study Three: An Unexpected Pregnancy

The third case presented here for analysis was selected from the research project Disability, immigration and multigeneration: intersecting factors in child protection cases, which is currently ongoing at the time of writing. This project was in many ways inspired by the first two, acting as an extension to pursue further themes that emerged but were not adequately explored in our earlier publications, such as the multi-generational effects of custody deprivation and the factor of immigrant parents who were often implied in various ways to be disabled. This project employs a case study methodology as well as semi-structured interviews with parents and legal professionals.

The case presented here concerns a single woman in her late 30s with a number of diagnoses, including mild ID and autism. The case unfolded in late 2019. Two notifications to child protection were made about the mother by healthcare and support agencies after she presented herself at the emergency ward of a hospital complaining of abdominal pain. Her pregnancy was diagnosed by staff of the emergency ward, after which she shortly gave birth. The rights protection officer for disabled people of this region of Iceland contacted the second author, who acted as the mother’s lawyer and advocate. Child protection’s plan was to immediately place the infant into temporary care, due to the mother’s supposed lack of ability and the maternity ward staff’s view that the mother presented a danger to the child.

### 2.4. Collective Case Study Methodology

The particular methodologies and the timeframes of the case studies vary, but the focus on parents with intellectual disabilities and their struggles with the child protection system remain the same. Our aim is to produce a synthesis of this body research to help to identify the factors that persist over time and allow these difficulties to continue. Following the lead of Hyett, Kenny and Dickson-Swift [39], we see instrumental case studies as useful in order to shed light on specific issues, whereas the collective refers to cases “*studied as multiple, nested cases, observed in unison, parallel, or sequential order*” ([39], p. 2). They advise researchers who use this collective case study approach to “*seek out what is common and what is particular about the case. This involves careful and in-depth consideration of the nature of the case, historical background, physical setting, and other institutional and political contextual factors*” ([39], p. 2). The cases we have purposefully selected share commonalities in that they concern parents or a parent with ID in Iceland who became a concern for the child protection system in the context of the maternity ward setting. The cases we selected exemplified many of the patterns we noted in our collective research; we also selected cases for which we had particularly detailed information. Following Boblin et al. [40] we have engaged in a form of triangulation in which “*varied sources of data are collected and analysed to obtain multiple perspectives and points of view to obtain a holistic understanding of the phenomenon being researched*” ([40], p. 1270). Triangulation is arguably problematic when the data are drawn from different research projects. However, Diefenbach [41] argues that is part of the nature of what triangulation is: “*data sources (e.g., data collected from different persons, or at different times, or from different places), using different methods (e.g., observation, interviews, documents, etc.), using different researchers, applying different theories, and using different types of data*” ([41], p. 882) which opens the door for numerous research possibilities.

### 2.5. Accounting for Bias

Bias arguably plays a role in all qualitative research in general and which has engendered criticisms as well as counter-arguments. There is not adequate space to present a comprehensive overview of the issues, but we are sympathetic to the position advocated by Diefenbach [41] who contends that bias will always be an inherent part of qualitative research, in terms of individual scholars, their positionalities and political or ethical orientations: “*There is no such thing like value-free or neutral social sciences and it is simply not possible to distinct social research and theory from social practice*” [41] (p. 889). Diefenbach contends that since it is impossible to remove the human factor from qualitative research, the issue then becomes to make this clear and its implications for methods and analysis.

A critical disability studies perspective always implies the position that the researcher is working towards the interests of disabled people, but that this must be made clear, “*stating clearly their ontological and epistemological positions and ensuring that the choice of research methodology and data collection strategies are logical, rigorous and open to scrutiny*” [42]. Stevenson contends [43] that researchers in the emancipatory research paradigm make a commitment to stand alongside oppressed populations and to work as co-participants committed to the study of change ([43], p. 38). The third author, after two decades of working with families headed by parents with intellectual disabilities, purses this advocacy and emancipatory model of research. The second author works as a legal professional and advocate on behalf of disabled people. The first author originally engaged in this research from a more distanced perspective, working at the intersection of anthropology and disability studies but not in the area of disability and parenting issues. However, in terms of full disclosure all authors have played roles, to varying degrees, as advocates for some of the parents in our body of research, either through providing assessments for the parents and their lawyer, as advocates for parents in the courts, or providing other forms of social support.

### 2.6. Ethical Review

The first project (2001–2003) was notified in 2000 to what was referred to as ‘tölvunefnd’ (no. 2000/796) under the auspices of the Icelandic Data Protection Authority (Persónuvernd), which was the only existent body at the time in Iceland that oversaw research practices. The project and its methodology was not deemed to be in breach of ethical research practices. The second project (2014–2016) received ethical review and approval from the Icelandic national bioethical committee, Vísindasiðanefnd, in 2014 (no. 14-062). The third project (2020–2022) was submitted for commentary to the Ethics Committee of the University of Iceland which determined that the study does not contravene the University’s Code of Ethics and had no reason to oppose the study (Vísindasiðanefnd Háskóla Íslands—7.4.2020). All names provided in the cases are pseudonyms, and certain details and dates have been altered or left imprecise in order to preserve as much as possible the privacy of the individuals concerned.

## 3. Findings

As the result of our collaborative case study analysis of three research projects, we have identified three major themes, and a number of related sub-themes, pertaining to the experiences of parents with intellectual disabilities in the maternity ward setting in Iceland. We contend that these themes interrelate and are conjoined due to the factors of structural and obstetric violence pertaining to parents with intellectual disabilities and a weak connection to the changing human rights environment. The major themes are Poor working practices, Lack of trust, and Selective focus. Evidence of these themes can be found in each research project spanning a twenty-year timeframe despite a changing social and human rights environment in Iceland concerning disability.

### 3.1. Poor Working Practices

A major theme we identified in all three cases are Poor working practices. Some instances are suggestive that basic administrative mismanagement and incompetence are responsible, but others we contend arise as the result of the factors of disability and structural violence. One sub-theme is the persistent lack of knowledge and professionalism as to how to react to and support parents with intellectual disabilities. Another related sub-theme is the poor information provided to families and their supports, and the alignment of the views of the professionals.

#### 3.1.1. Lack of Knowledge and Professionalism

In Case One the parents, both of whom have intellectual disabilities (Halli and Anna, pseudonyms), were forced to stay in the hospital for eleven days after the birth of their child while the hospital staff, social services and child protection worked out what to do. This ultimately resulted in the couple permanently losing custody of their child some six weeks later. A plan was initially implemented by social services to support the parents, however a key social worker was on vacation when Anna went into labour prematurely, resulting in what the social worker described as an “*absolute shambles*,” as the hospital staff appeared to panic when faced with the prospect of the risks they perceived in discharging the parents and notified child protection. After a brief ten-day stay at a training home were the new parents could be observed, the couple were forced to sign a temporary custody deprivation order. A paediatric nurse with experience in supporting parents with intellectual disabilities, and who met the mother and was aware of the circumstances, commented upon how the case was handled:

“*I consider a three-month period in the training home the minimum time required for these parents [and] only then should a decision be taken on the way forward…A big part of my work is to prevent staff from overreacting because parents have learning difficulties; an overreaction that would not happen if it had been other parents.*”

Anna’s mother argued that the lack of proper support tailored to meet the couple’s needs appeared to make custody deprivation a forgone conclusion: “*With a training period being both short and inadequate they never stood a chance*”.

In Case Two, which occurred around a decade later, a notification to CP was made concerning similar fears by healthcare staff about the child rearing abilities, or more accurately the lack thereof, of a couple with intellectual disabilities (Daníel and Dísa, pseudonyms). In this case, after the healthcare services reported Dísa’s pregnancy to child protection, the assigned CP worker required that the couple partake in certain training activities for expectant parents. This in and of itself was not unusual, though some of the specific activities were. For example, the couple had to carry around training dolls that they had to treat like infants, which they found humiliating and degrading. Both Daníel and Dísa each had their own doll, despite only having one child. Additionally, the father’s doll repeatedly malfunctioned which caused the parents undue stress and anxiety. Other practices departed from the expected norms. When the child was born prematurely, CP decided that the assessment should be carried out in the maternity ward. The parents found it very uncomfortable to undergo a parenting assessment under these circumstances and even some of the maternity ward staff questioned the legality of keeping the parents in the maternity ward against their will while the assessment was conducted.

The third case, which unfolded in late 2019, concerns a single woman in her late 30s (Gerður, pseudonym) with a number of diagnoses, including mild intellectual disability and autism. Two notifications to child protection were made about Gerður by healthcare and support agencies after she presented herself at the emergency ward of a hospital complaining of abdominal pain. Gerður’s pregnancy was diagnosed by staff of the emergency ward, after which she shortly gave birth. Child protection’s plan was to immediately place the infant into temporary care due to Gerður’s supposed lack of ability and the maternity ward staff’s view that Gerður presented a danger to the child. A careful examination of the evidence documented by child protection to support their actions revealed sloppy or poor working practices. For example, in a report by a psychiatrist he attests that the mother has no history of psychosis nor disconnects from reality. However, in a summary of the report written by child protection a claim is made that she has a history of psychosis. In a report from a worker hired to observe the mother’s interaction with her child, the mother is described as not being able to take direction. Yet in a report written five days later by another worker it is stated that the mother “*takes guidance well*.” Gerður’s lawyer, after reviewing the case data, commented:

“*Nothing in her medical records supports their claim of incompetence of [Gerður’s] ability as a mother and her psychiatrist doesn’t see anything standing in the way of her being a mother with proper support*.”

#### 3.1.2. Poor Communication

In all cases there is evidence that communication between parents, and their families, with healthcare professionals and child protection workers was poor. Information that was provided was often false, misleading or partial and with key stakeholders excluded from meetings and the general flow of information. In Case One, meetings were held and decisions were taken without the parents’ full knowledge, and only limited or misleading information was communicated. Anna’s mother commented: “*We as a family felt ill-informed, at times intimidated and never given enough time to consider options or take decisions*.” Halli, the father, fully aware of the suspicions about their perceived inability to parent, sought to ensure that they were doing everything right:

“*Again and again we asked them if we were doing anything wrong and they responded everything was good and that all parents can make mistakes…We received no information about us doing anything seriously wrong. Neither ourselves nor our family were told that something was not good enough about our performance*.”

In Case Two, Dísa’s mother tried to support her daughter, knowing full well the working practices of child protection after having dealt with CP interference when she was a mother herself. However, despite the full support she gave her daughter, she was rarely listened to or even acknowledged: “*They never listen to me, it is like I was not there*.” The voices of the parents only became ‘heard’ with the help of a highly respected and well-known lawyer in Iceland who acted as an advocate on their behalf. Similarly, in Case Three, Gerður, the mother, when talking to a supportive staff worker complained that in the maternity ward she was only given messages about what she did wrong, but never shown exactly how to do things properly.

#### 3.1.3. Alignment of Maternity Ward and Child Protection Professionals

While there are some critical voices from healthcare professionals, lawyers and other advocates in the data, the data also reveal a strong tendency for the views of professionals to align to favour custody deprivation in the case of parents with intellectual disabilities. After the birth of the child in Case One, both parents and extended family members reported that the views and working methods of most of the professionals, from social services to child protection, to the hospital staff and even their own lawyer and advocate (Ombudsman for disabled people) appeared to align in support of the goal of custody deprivation. Halli’s father noted his surprise at how quickly the professionals, who were supposed to be their advocates, turned their backs on the parents:

“*I would have liked to see the lawyer stand with them as I expected any lawyer to stand with and represent their client. Instead he, like the Regional Ombudsman, worked with the other party against the couple*.”

Halli, the father, also commented upon this:

“*We feel like both the Regional Ombudsman and the lawyer failed us, they were mainly concerned about us signing the papers and finishing the case.*”

Anna’s mother noticed this as well, registering her surprise: “*It was so strange; it was as if our lawyer was representing the Child Protection Service and not my daughter and her partner.*”

In Case Two, both of the parents, Daníel and Dísa, were also subjected to the expected alignment of the professionals against them, as seen in the exclusion of the parents from the flow of information. In Case Three, this alignment occurred rapidly as evidenced in the form the narrative concerning Gerður’s inability to parent that was constructed shortly after birth. The maternity ward staff from the start argued that they did not trust Gerður with the child, claiming she knew nothing about infant care. These views were also echoed by child protection staff who were quickly called into the case. A child protection worker stated to Gerður’s lawyer that less stringent measures simply could not be taken due to the two notifications that were made by healthcare and support agencies. In her lawyer’s view, child protection had essentially reversed the burden of proof; Gerður was judged incompetent by the professionals on the basis, presumably, of her disability and the burden to prove competence was placed upon the mother right from the beginning. The alignment of the views of the professionals also worked against the advocates for the parents. Gerður’s personal spokesperson was repeatedly excluded from meetings and information sessions with child protection and healthcare professionals, including the parental assessment process, making it difficult to advocate effectively on behalf of her client.

### 3.2. Lack of Trust

A common theme throughout this body of data is the lack of trust. From the point of view of the professionals in the maternity ward setting this is articulated as a lack of trust in the parents’ abilities and fear that they will harm their child. From the point of view of the parents, this lack of trust is produced as the result of the amplification of any parenting mistakes they made, the pressures of the constant surveillance they were under and the perceived hostility they encountered on the part of professionals. Particularly in Case Two the initial trust needed to be built, as both parents experienced child protection interference in their lives when they were children.

#### 3.2.1. Under the Microscope

In Case One, the father Halli commented upon the pressures the couple felt due to the constant surveillance they were placed under. This was combined with the perception that the healthcare and child protection workers were primarily focused on documenting their mistakes, rather than providing proper guidance or acknowledging their strengths:

“*We were under the microscope all day. It was very uncomfortable to have someone keeping an eye on us all the time and mainly because we felt they were looking for our mistakes but not if we could do this*.”

Anna, the mother, confirmed this:

“*I found this difficult when others were watching me so closely and I was so frightened of making mistakes. Doing this on my own or with people I know very well, like my family, would have been easy*.”

This lack of trust in the parents appeared to extend toward family members as well, as Halli’s father noted:

“*While Halli and Anna took care of the baby the two staff members stood over them all the time. It was not enough for me to be there, I could sit and watch but I was not trusted to be alone with them*.”

In Case Two, both Daníel and Dísa’s prior childhood experiences of child protection and foster care left the couple upset and apprehensive about any CP involvement in their lives and which amplified their constant fears the possibility of custody deprivation. Dísa simply stated: “*I just don’t trust them*.”

#### 3.2.2. The Amplification of Parenting Mistakes

When mistakes did happen, as common particularly with first-time parents, in the context of parents with ID in the maternity ward setting these missteps resulted in a variety of harsh measures, such as notifications to child protection or hostile admonitions from healthcare workers. In Case One, Halli argued that all missteps they made were exaggerated. As evidence he cited a report from the hospital which stated: “*an example of their inadequacy is that they don’t sense how much milk the child needs each time nor how often it needs to drink*.” He continued to express his frustration about these attitudes:

“*It seemed to us like it had become a crime to ask questions, misunderstanding always followed. I often asked questions like how much should the child get and once when the child was moving I asked if it needed to be fed. I didn’t know anything about babies but the way we were treated I feel like they expected us to be born into the parental role*.”

Anna’s mother noted her surprise as some criticisms that were made toward about his parenting skills:

“*They claimed Halli was tired feeding the child in his arms and put the child into the cot supporting the feeding bottle by the duvet… They made this out to sound so serious that I thought something terrible had occurred… In earlier days it was a common practice feeding children in this manner and maybe he hadn’t been taught otherwise*.”

In Case Three, Gerður’s lawyer described how angrily healthcare workers were toward the mother’s lack of knowledge of infant care and any missteps she made. Gerður’s lawyer recalled the scene she encountered at the hospital in a meeting with healthcare and child protection workers:

“*At 11 AM I’m at the hospital where there is a meeting with CP and two doctors from the maternity ward. The doctors look upset and say that they don’t trust Gerður for one second with the child. They claim that she can’t hold it, she almost dropped it and that she has shown no interest in the child. She didn’t know how to feed it or change it. I tried to get them to understand that having a baby was a shock to anyone let alone if you didn’t know you were pregnant and had ID or autism*.”

### 3.3. Selective Focus

In all cases there is a notable selective focus on the factor of disability on the part of healthcare and child protection. The factor of ID itself is at times either selectively ignored, or exaggerated to the point where all parenting lapses or missteps are attributed to the impairment and which overlooks or ignores the relevant context in which the incident occurred. The parents in these cases, as in the cases in our broader body of research, have some form of intellectual or development disability. This status is often implicitly or explicitly invoked to explain any and all behaviours of the parents that are seen as troubling. The parents are keenly aware of how negatively they are viewed by the healthcare and child protection system, and the emphasis that is placed upon their impairments. In Case One the father Halli made this clear: “*often I feel like they regard me as an idiot that doesn’t know what is going on. I feel like they speak down to both Anna and myself*.” Anna added: “*I agree*.” The attitudes on display in the maternity ward setting appeared to confirm their views. A healthcare worker basically infantilized the couple in an interview, stating:

“*Both of them were very caring, that was not the problem. However, we soon recognised how they were like small children themselves who could not assess their child’s needs accurately… There were many things in their behaviour that made us feel like they were children looking after a child*.”

These views appeared to extend beyond the healthcare workers to other professionals. For example, during Anna’s pregnancy a worker from social services encouraged her to have an abortion even though there was no serious health issue with regard to the viability of the foetus. In Case Three, a significant amount of text in reports about the mother is devoted to negative descriptions of her physical appearance and a mention was also made that she spent most of her day in the hospital watching “cartoons” without any qualification as to the relevance of these observations about the matter at hand which was assessing parental competence in the context of a disabled mother who just experienced an unexpected birth.

It is notable how at times the factor of ID is selectively invoked, and the relevant context downplayed or ignored. Throughout the narratives of the case data the assessments about the parents’ lack knowledge and skills is routinely divorced from the fact they are first time parents. This is particularly significant in Case Three where the mother did not know she was pregnant until she went into labour and no seeming allowance is given for her lack of knowledge and preparation in infant care. An analysis of the reports produced by child protection about the mother Gerður suggest that an emphasis was placed upon her lack of awareness of her pregnancy as one key factor upon which to make a case for her inability to be responsible for a child. In Case One, Anna’s aunt went to visit her niece in the hospital and was surprised to learn that she had been discharged and the child taken into care by child protection. Upon asking why Anna’s parents were not informed of this, Anna’s aunt was told by a healthcare worker: “*Because they are consenting adults and do not live with their parents*.” Anna’s mother sarcastically commented: “*Suddenly they were considered fully able*.”

## 4. Discussion

We have employed a collective case study methodology to re-examine the data from three case studies drawn from three research projects in Iceland spanning a twenty-year period. The cases all concern parents with intellectual disabilities and their difficult experiences in the maternity ward setting. The cases were selected in part because of our detailed knowledge about them, but also because they exemplified larger patterns we have become aware of as the result of our collective research and professional experience in Iceland. Our aim is to identify, through a longitudinal and comparative framework, what these patterned difficulties are with the goal of contributing to understanding why they persist despite a changing disability rights environment. We have identified three major themes—Poor working practices, Lack of trust, and Selective context—and a number of related sub-themes that are common to their collective experiences. Parents with disabilities, and intellectual disabilities in specific, are not the only parents to lose custody of their children to child protection. However, the disproportionate rate at which these parents lose custody of their children, as noted in the international literature [2,3,4], warrants further attention. As is the persistence of these outcomes pertaining to custody deprivation.

The key to our analysis is the framework of structural violence, drawn from the work of Galtung [21] and extended with the concept of obstetric violence [23,24,25,26,27,28,29] in light of our focus on the maternity ward setting. The direct act of custody deprivation is supported by structural and cultural factors which limit the life chances and opportunities of these parents in general, and their ability to maintain the custody of their children in specific. The marginality and isolation of the parents in Cases Two and Three was significant, rendering them particularly vulnerable when the views of the professionals aligned against them. Case One was different in that both parents had the support of their extended families. Yet even this did not make them, or their families, immune to forms of structural violence. We provided examples of where family members and advocates were also ignored and excluded from the flow of information. In Case One, so convinced of the parents’ inabilities even the grandfather was not trusted to be alone with the parents and his grandchild. The persistent lack of knowledge about supporting parents with intellectual and developmental disabilities, the selective focus on context when assessing the parents, and lack of trust continued to result in child protection rapidly choosing custody deprivation as a solution. The fears and exaggerated views of parenting with a mild or even borderline ID appeared to play a significant role, from focusing on weaknesses instead of strengths, assuming the parents are a risk from the outset despite a lack of evidence, to the apparent panic that seemed to take hold in the maternity ward.

The analysis of structural violence allows us to pay closer attention to the interconnections between the act of child removal itself, the structurally-based disempowered positions of the parents (and by extension their families), and the negative cultural attitudes toward parenting with ID. Cultural violence is an important lynchpin in Galtung’s conceptualization of violence. These ideologies explain or legitimate the actions of child protection in cases where custody deprivation appears to be predicated primarily upon the factor of disability. Within the obstetric violence literature, the hostile and negative attitudes toward mothers who are poor, or indigenous or of ethnic minority status are evident [24,25,26,27] and maternity ward staff may not look favourably upon their parenting abilities. But in our estimation custody deprivation on the basis of these marginalised statuses alone did not seem to be pursed to the same degree and intensity as with parents with intellectual disabilities. The persistent hostility displayed toward the very idea of these parents being parents by healthcare and child protection professionals was notable in the data in all three cases. Eugenic inspired policies and practices have been pursued against disabled people across the North Atlantic, including Iceland [9], in the past but there is some evidence that these attitudes persisted into the early 21st century. For example, the parents in Case One were advised to abort their child, even though there was no medical basis for this. We have seen some limited evidence of a less extreme nature of this kind of thinking in more recent years. In one example Dísa, the mother from Case Two, was pressured to use an implanted form of contraception. In Case Three, this form of contraception (Is. getnaðarvarnarstafur) was implanted in the mother in the maternity ward. We have pondered if these are but a more socially acceptable form of the prevention of parenting with ID in an era in which forced sterilization and coerced abortion are illegal and institutionalization has generally fallen out of favour.

The contextual background of our re-analysis of the data concerns the changing social and human rights environment in Iceland in regard to the rights of disabled people. As Iceland signed (2007) and ratified (2016) the United Nations Convention on the Rights of Persons with Disabilities (CRPD) we wanted to know if there was any evidence for the influence of the CRPD or a general language of disability rights in the case data. In the Case One, which pre-dated the CRPD, there is no evidence of the articulation of a discourse of human rights in general or in regard to disability in specific. In both the post-signing and post-ratification environment in Iceland, the CRPD does not seem to have been incorporated into CP working practices in our case data. There appears to be a persistent view within CP that the right to reasonable accommodation is based on the assumption that the rights of the parents and the rights of the child are in contradiction. If everything is narrated through the perceived rights of the child (not to have a disabled parent), the rights articulated within the CRPD become moot, as are any procedural guidelines for working with parents with ID. Child protection in Iceland remain hostile toward the idea of parenting with an intellectual disability. This can be seen in our case data but also in the operational definition of parental neglect in Iceland (2012). The definition holds that, among other things, leaving a child in the temporary care of someone deemed to be unfit or incompetent is considered to be a form of neglect, and this includes leaving children in the care of someone who is developmentally impaired (Is. þroskaskert) [31].

During the course of our analysis of custody deprivation cases from 2002 to 2016, there were negligible references to human rights and parenting in relation to disability. Since this time the second author, who works as a legal professional in Iceland, could think of very few custody deprivation cases which invoked the CRPD; one example being the Icelandic Supreme Court case 435/2017 which ruled that the interests of the child, supported by the Icelandic Constitution, Icelandic Child Protection Act and the UN Convention on the Rights of the Child, take precedent over the rights of the parents and Article 23 of the CRPD. The influence of the CRPD on the ideologies and working practices in the child protection system continue to remain weak, though further research would need to be done to determine if this is so, and why. At the time of writing, the national level child protection agency in Iceland, Barnaverndarstofa, makes no reference to the CPRD, and this is significant as one of their purposes is to guide the work of the local level municipal child protection committees.

Caution needs to be employed toward the findings due to the limited sample size, though the cases selected serve as exemplars of larger patterns we have noted in our collective research and professional experience in Iceland. Further work still needs to be done in order to strengthen our claims. Despite our findings, the effects of the changing human rights context in Iceland concerning disabled people and child protection remains to an extent unclear and continues to be an area which calls for further investigation into why this is. Further research into the education and training of child protection workers, and the organizational culture within CP, would be another way forward in order to understand why these patterns in Iceland persist.

## 5. Conclusions

We contend that our collective case study re-analysis suggests that patterned forms of structural violence persist against parents with intellectual disabilities who come into contact with the child protection system in Iceland, particularly in the maternity ward setting. The patterns have shown to be resilient, despite the cases here occurring at different points within a twenty-year time span and set against the background of a changing legal and human rights environment in Iceland. The signing and ratification of the CPRD appears to have limited effects in this area. Finally, we contend that structural violence, and obstetric violence in particular, remains a useful analytical framework from which to understand the experiences faced by these parents and could also be useful for advocacy purposes in drawing attention to the issues and lobbying for meaningful systematic change.

## Data Availability

The data presented in this study are available on request from the corresponding author. The data are not publicly available due to ethical and privacy issues.
